# Comparative Analysis of Proanthocyanidin Metabolism and Genes Regulatory Network in Fresh Leaves of Two Different Ecotypes of *Tetrastigma hemsleyanum*

**DOI:** 10.3390/plants11020211

**Published:** 2022-01-14

**Authors:** Erkui Yue, Yuqing Huang, Lihua Qian, Qiujun Lu, Xianbo Wang, Haifeng Qian, Jianli Yan, Songlin Ruan

**Affiliations:** 1 Institute of Crop Science & Ecology, Hangzhou Academy of Agricultural Sciences, Hangzhou 310024, China or Yueerkui0501@163.com (E.Y.); huangyq@zju.edu.cn (Y.H.); ddmqlh@hotmail.com (L.Q.); chayesuo2008@163.com (X.W.); 2 College of Environment, Zhejiang University of Technology, Hangzhou 310014, China; hfqian@zjut.edu.cn; 3 Agricultural and Rural Affairs Guarantee Center, Hangzhou Agricultural and Rural Bureau, Hangzhou 310020, China; luqiujun@163.com

**Keywords:** *Tetrastigma hemsleyanum*, proanthocyanidins, ecotype, *LAR*, *ANR*

## Abstract

*Tetrastigma hemsleyanum* Diels et Gilg is a rare and wild medicinal resource. Metabolites, especially secondary metabolites, have an important influence on *T. hemsleyanum* adaptability and its medicinal quality. The metabolite proanthocyanidin (PA) is a polyphenol compound widely distributed in land plants, which can be used as antioxidants and anticancer agents. Here, we discovered that three types of PA accumulated in large amounts in purple leaves (PL), but not in green leaves (RG), based on widely non-targeted metabolomics. In addition, we further found that catechins and their derivatives, which are the structural units of PA, are also enriched in PL. Afterwards, we screened and obtained five key genes, *DNR1*/*2*, *ANS*, *ANR* and *LAR* closely related to PA biosynthesis through transcriptome analysis and found they were all highly expressed in PL compared to RG. Therefore, observed the regulatory relationship between the main compounds and genes network, and the PA metabolism regulatory pathway was complicated, which may be different to other species.

## 1. Introduction

Traditional Chinese medicinal plants are a vital source of polyphenols, which are believed to be greatly beneficial for cancer chemoprevention [1,2]. *Tetrastigma hemsleyanum* Diels et Gilg, included in the genus Tetrastigma belonging to the family Vitaceae, is a plant mostly distributed in southern China that is used to make the traditional Chinese medicine called “Sanyeqing” [3]. A previous study reported that the leaves of *T. hemsleyanum* were rich in phenolics [4]. The proanthocyanidins (PAs), which are the second most abundant plant polyphenols, are a large class of polyphenol compounds widely present in a variety of plant parts [5,6,7,8,9,10], such as fruits, vegetables, grains, nuts, and leaves. Structurally, PAs are oligomers or polymers of flavan-3-ols that contain catechin or epicatechin [11,12], and they are the main components of dietary polyphenols.

PAs are known to be important natural anti-aging and anti-cancer products; they scavenge free radicals in cancers of various organs, such as ovarian cancer, colorectal carcinoma, and skin cancer [13,14,15,16]. Furthermore, as a natural antioxidant, PAs extracted from blueberry leaves can prevent hepatitis C virus replication [17]. Subsequently, more scientific studies have shown that PAs do have strong medicinal value. The regulatory gene networks involved in the molecular mechanism of PAs have been fully discovered and explained in many land plants [10,18,19,20,21]. As a matter of fact, PA synthesis is involved in flavonoid pathways, meaning that the isoflavones, flavonols, and anthocyanins are also a prerequisite for the synthesis of PA [20]. Furthermore, the key enzymes in the specific production pathway of PA include leucocyanidin reductase (LAR) and anthocyanin reductase (ANR) [20,22,23]. It is well known that both monomeric catechin and epicatechin are involved in the biosynthesis of PA, and the accumulation of catechin and epicatechin monomers requires the participation of LAR [21], which reduce the leucoanthocyanins (LAs) to form catechins. The LAR gene was first discovered in the leguminous plant (*Desmodium uncinatum*) [24]. LAs are generated by the reduction of dihydroflavonol by dihydroflavonol reductase (DFR) [25,26]. As reported previously, LAR expression was found to be related to the synthesis of catechins and PA in the apple. It contributes to PA synthesis in fruits, and the tissue- and time-specific regulation of LAR determines the accumulation and composition of PA during grape development [27]. In addition, in the biosynthesis of epicatechin, first, anthocyanin synthase (ANS) converts LAs into anthocyanins and then ANR converts anthocyanins into epicatechins [20,28]. The *ANR* gene has, thus far, been successfully isolated and cloned from *Arabidopsis*, tea, apple, alfalfa, and several other plants [28,29,30,31,32], and the stable expression of ANR in various plants is related to the accumulation of PAs. Further, catechins and epicatechins can be polymerized to different polymerization degrees to form various PAs [29]. 

*T. hemsleyanum* and *Vitis vinifera* have a relatively close evolutionary relationship. However, whether the copy numbers of LAR, ANR, DFR, and ANS genes in *T. hemsleyanum* are consistent with those of other species is ambiguous. Moreover, it is unclear whether there exists a similar molecular pattern that regulates PA. We selected two ecotypes to identify metabolite alterations and found a much higher content of PAs in the PL than the RG plants. Therefore, we obtained two ecotypes, RG and PL, to explore the differences in their PA metabolic pathways and gene regulatory networks. Clarification of the metabolic regulation pathway of PAs in *T. hemsleyanum* is conducive to the subsequent development and utilization of PAs and lays a sound foundation for the development of new formulations of anticancer drugs based on Chinese herbal medicines. As such, the development prospects of PAs are huge.

## 2. Results

### 2.1. Metabolic Alterations between the RG and PL Ecotypes

Orthogonal partial least-squares discriminant analysis (OPLS-DA) is a supervised discriminant analysis statistical method that differs from the principal component analysis (PAC) method. To discriminate the metabolic profiles of the relationship between metabolite expression and sample categories, we performed clustering analyses based on OPLS-DA with variable importance in projection (VIP). The differential metabolites were visualized and filtered through the OPLS-DA S-plot.

**Figure 1:**
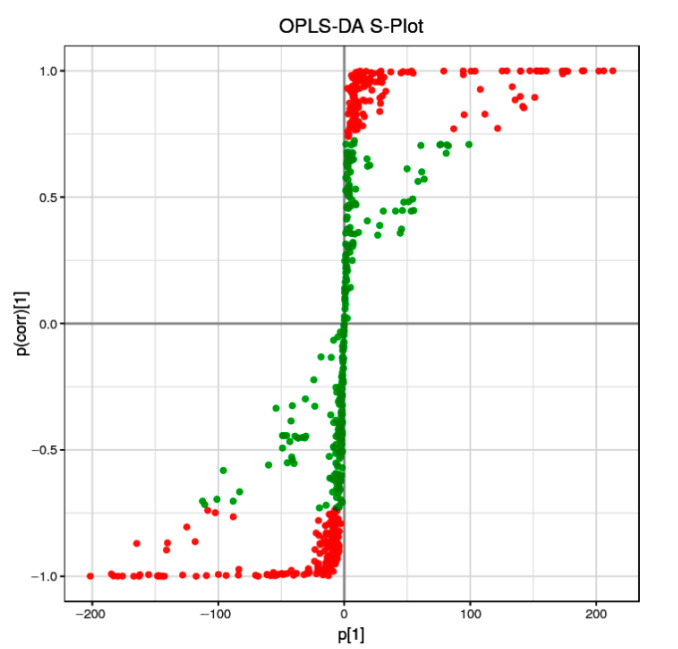
OPLS-DA S-plot of RG versus PL samples. Each point represents a metabolite, red color indicates metabolites with VIP ≥ 1, green indicates metabolites with VIP < 1.

We discovered that nearly half of the metabolites (green points, VIP < 1) were distributed not far from the origin of the coordinate. Meanwhile, the other points (red points, VIP > 1) were further from the origin of the coordinate (Figure 1), suggesting a greater confidence level of each metabolite to the clustering observed in the score plots of OPLS-DA between the two sample groups (RG and PL).

**Figure 2:**
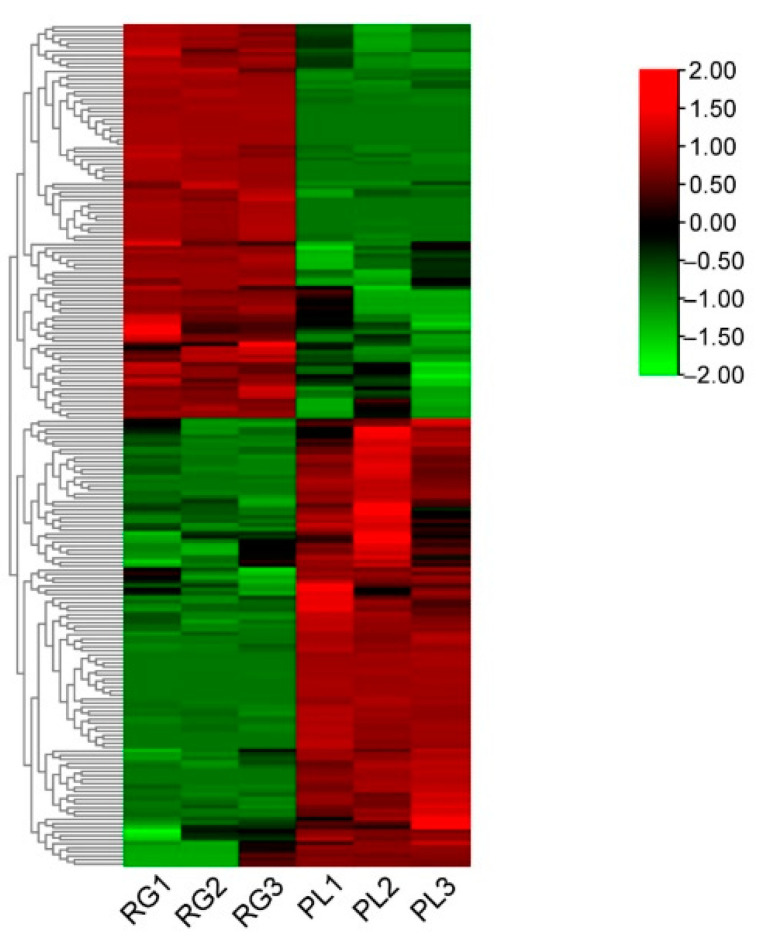
The heat map of the leaf relative levels of each metabolite and the unsupervised hierarchical clustering from independent biological replicates in *T. hemsleyanum*. The metabolites relative content in low and high are shown in green and red, respectively.

In order to better delineate the changing rule of metabolites in leaves between the RG and PL ecotypes, over 2-fold of metabolites were chosen and normalized, we then obtained a cluster heat map. The results showed that 53% of the metabolites were much higher in PLs than RGs, while 47% were significantly lower in PLs than in RGs (Figure 2). Further, these metabolites mainly refer to flavonoids, flavonols, catechins, procyanidins, and organic acids (Table S1). 

### 2.2. Proanthocyanidins (PAs) Were Highly Enriched in the PL Ecotype, but Not in the RG

**Figure 3:**
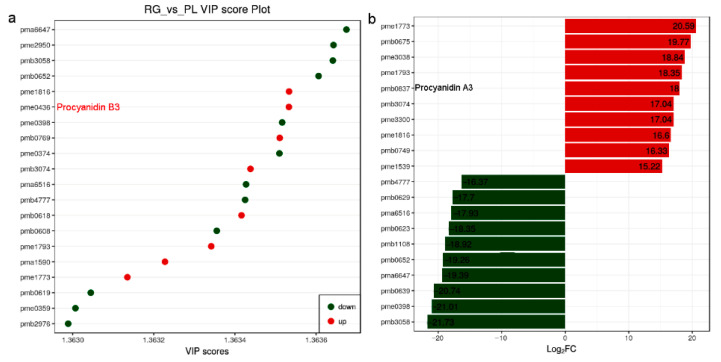
Metabolites with a VIP value greater than 1.363 and the distribution of differential metabolites with a multiple of 15 times or more, and in *T. hemsleyanum*. (**a**) Variable importance in projection (VIP) value (>1.363) used to screen differential metabolites. It is generally believed that variables satisfying both *p* < 0.05 and VIP > 1.0 are differential metabolites; (**b**) After qualitative and quantitative analysis of the detected metabolites, combined with the grouping of specific samples, compare the fold change of the quantitative information of metabolites in each group (log2FC > 15).

As we all know, PAs are flavonoid polyphenol compounds that are widely present in nature and have a variety of biological activities. Subsequently, the PA and its upstream metabolites attract our attention due to their health benefits in humans [33]. We found 8 metabolites were up in PL but 12 were down according to the VIP score plot. Among them, pme0436 represented procyanidin B3, its VIP value over 1.3635 suggesting it might have a greater impact on the model. In addition, compare the fold change of the quantitative information of metabolites in each group; procyanidin A3 (pme0837) value of log2-fold change (FC) (PL/RG) was 18, meaning the PA was more abundant in PL (Figure 3; Supplemental Table S1).

**Figure 4:**
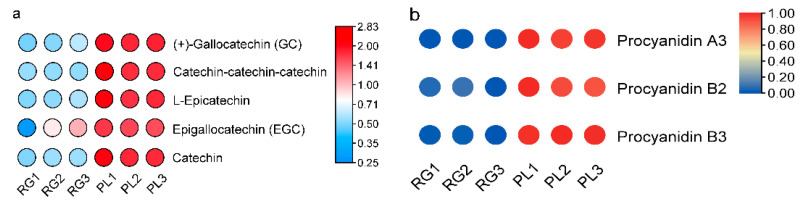

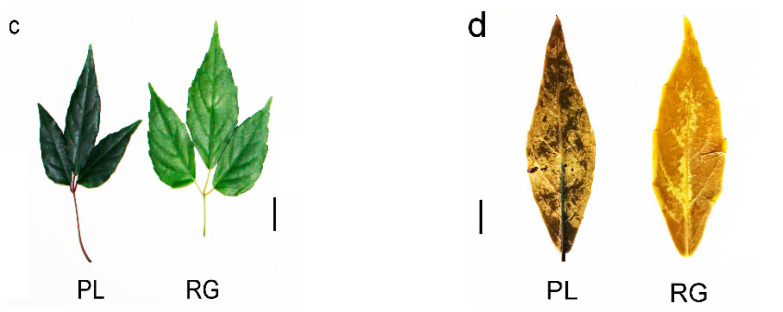
Predicted annotations of specific metabolite PAs detected in the leaf of *T. hemsleyanum*. (**a**) The content of catechins and its derivatives in the leaves of RG and PL (VI P > 1); (**b**) The content of PAs in the leaves of RG and PL (VIP > 1); (**c**) The phenotype of leaf color in the different ecotypes of *T. hemsleyanum* (left panel: PL; right panel: RG); (**d**) Comparison of PAs content in PL and RG ecotypes of *T. hemsleyanum* through DMACA staining method.

Generally speaking, PAs are oligomers or polymers composed of (+)-catechin and (−)-epicatechin, and their main structural components are flavan-3-ol. Our results showed the PLs were rich in catechin and epicatechin and their derivatives, as well as in PA (Figure 4a). The PA mainly included A-and B-type PA (Figure 4b). Moreover, the PL leaf color was a deeper green than the RG leaf, meaning that the PA content in PL might be higher than in RG (Figure 4c). Thus, we utilized DMACA staining to analyze the accumulation of PA in *T. hemsleyanum*. The PL leaves color were stained blue, indicative of PA, but no blue material PA was seen in the RG leaves, illustrating that the PA content in PL leaves is much higher compared to that in RG leaves. (Figure 4d).

### 2.3. The Differential Genes Analysis Based on Transcriptome

**Figure 5:**
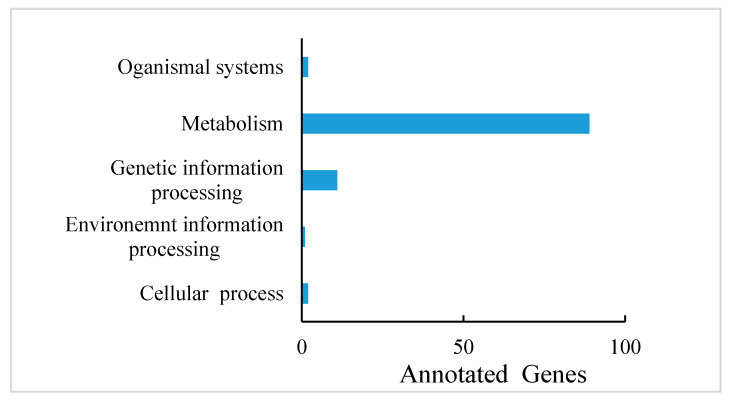
Differentially expressed gene annotations in the KEGG pathway of *T. hemsleyanum*.

**Figure 6:**
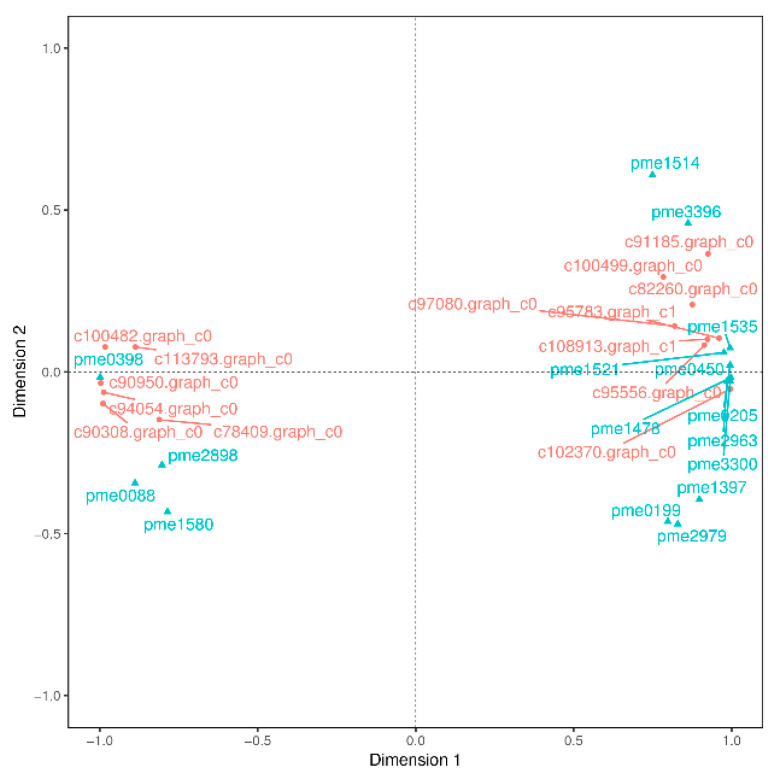
Canonical correlation analysis of differential genes and metabolites in *T. hemsleyanum*. The red color represents the gene ID, and the blue indicates its meta name. The genes annotations information as follows: c78409.graph_c0 (vinorine synthase-like), c100499.graph_c0 (2OG-Fe(II) oxygenase superfamily), c95556.graph_c0 (flavanone 3-hydroxylase-1), c102370.graph_c0 (flavonol synthase), c94054.graph_c0 (cytochrome P450 98A2), c90308.graph_c0 (flavonol synthase), c95783.graph_c1 (Flavonoid 3’,5’-hydroxylase), c91185.graph_c0 (dihydroflavonol 4-reductase-like), c90950.graph_c0) (stilbene synthase 3), c113793.graph_c0 (shikimate O-hydroxycinnamoyltransferase isoform X1), c82260.graph_c0 (cytochrome P450 98A2), c108913.graph_c1 (Dihydroflavonol 4-reductase), c100482.graph_c0 (spermidine hydroxycinnamoyl transferase-like); the metanames represents as follows: pme1535((+)-gallocatechin (GC)), pme0398 (chlorogenic acid), pme2898 (dihydromyricetin), pme0088 (luteolin), pme1580 (eriodictyol), pme0450 (L-epicatechin), pme1514 (epigallocatechin (EGC)), pme0205(catechin), pme3396 (fustin), pme1521 (dihydroquercetin), pme1478 (myricetin), pme2963 (dihydrokaempferol), pme3300 (tricetin), pme1397 (pelargonidin), pme0199 (quercetin), and pme2979 (dihydrochrysin).

PA are known to occur at higher levels in PL, but how and which genes control their synthesis and metabolism in *T. hemsleyanum* have not been reported. First, we classified the annotation results of the differentially expressed genes in terms of the types of KEGG pathways. The result displayed that most of the genes participated in metabolic processes (Figure 5). In addition, to further explore the relationships between genes that regulate PA metabolism, we carried out a canonical correlation analysis to reveal the differential genes and metabolites in the correlation network; the four regions are distinguished by crosses in Figure 5. In the same region, the farther away from the origin, the higher the correlation. Flavonols that were related to PA synthesis, such as pme1521, pme2963, pme1196, pme1478, pme0199, and pme2898 were correlated with PA synthesis genes, including c95556.graph_c0, c102370.graph_c0, c108913.graph_c1, and so forth. These structural and regulatory genes are likely to take part in the flavonoid metabolic pathway (Figure 6).

**Figure 7:**
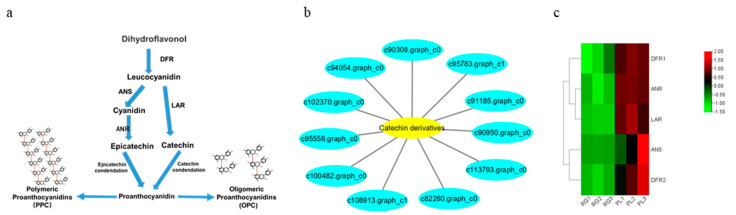
Analysis of key gene differences in the synthetic pathway of PA in *T. hemsleyanum*. (**a**) The synthetic pathway diagram of PA ubiquitous in plants; (**b**) The genes network related to the metabolism of catechin and its derivatives; (**c**) Cluster analysis of DEGs related to PA. Genes with a high expression in the RG vs. PL are shown in red and green, respectively.

**Figure 8:**
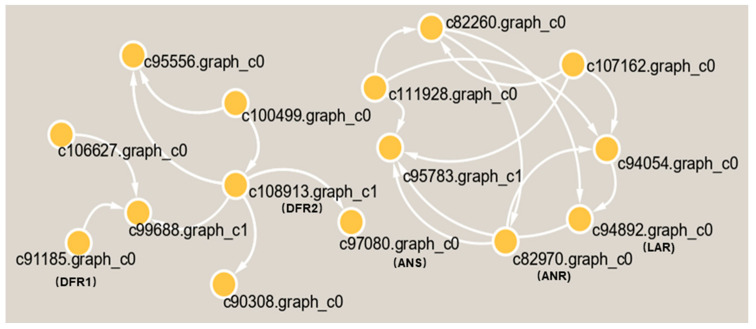
The interaction network of differential proteins in the metabolic pathway of PA in *T. hemsleyanum*.

Subsequently, we found 11 genes that were closely related to catechin and epicatechin derivatives according to their common PA synthesis pathway, and *LAR*, *DFR1*/*DFR2*, *ANR*, and *ANS* were highly expressed in PL (Figure 7). To further investigate their relationships within the protein–protein interaction (PPI) networks (Table S1), we constructed an interaction network of genes related to PA metabolism (Figure 8). This demonstrated that PA formation is an integrative and complicated regulation network. 

## 3. Discussion

PAs are the second most abundant polyphenolic compounds in plants [10], and more and more studies have shown that they are related to a reduced risk of cardiovascular disease, cancer, and Alzheimer’s disease. Further, they can also improve nutrition, prevent ruminant swelling, and enhance soil nitrogen retention [13,34,35,36,37,38]. We found them to be highly accumulated in a particular variety of PL (Figure 3 and Figure 4), meaning that PAs could represent important active agents of Chinese herbal medicine, and may be employed as useful therapeutic agents after extraction. 

In this research, we analyzed the accumulation of PAs in the leaves of the Chinese traditional herb “Sanyeqing” and noted the expression characteristics of genes encoding *DFR1*/*DFR2*, *ANR*, and *LAR* in two varieties. Afterwards, we used liquid chromatography tandem mass spectrometry LC-MS/MS, non-targeted metabolomics [39], and a staining method to detect the PA content of the leaves (Figure 3 and Figure 4b), after which the expression of *LAR*, *DFR1*/*DFR2*, *ANR*, and other genes were analyzed through transcriptomic data [39]. Our results showed that these genes were highly expressed in PL (Figure 7c) and might contribute to the biosynthesis of PA and the monomeric catechins and their derivatives in *T. hemsleyanum* (Figure 4a). Similarly, PAs were found to be present in grape leaves; however, they were highly accumulated in mature leaves and quite low in the young leaves of the tea plant [20]. Previously, we reported that the PL variety is also rich in anthocyanins [39]. We further found that PL is not only enriched in anthocyanins but also demonstrated that more LAs are converted into anthocyanins by ANS and this may result in fewer LAs forming PA. However, interestingly, the level of PAs in PL leaves increased rather than decreased, suggesting that more anthocyanins were converted into PA by ANR, and the high expression of *ANR* (Figure 7c) seems to support this. Our findings illustrate that even if the anthocyanins content were elevated, it would not affect the synthesis of PA in leaves of *T. hemsleyanum*. 

From another aspect, the gene annotations in the KEGG pathway also showed that most of the genes are related to plant metabolism (Figure 5). LAs are intermediate metabolites that participate in the synthesis of downstream anthocyanin and epicatechin and are involved in the synthesis of PA (Figure 7a) [39]. These processes involve two key genes, *ANR* and *LAR*, and we initially found that only one copy each of *LAR* and *ANR* may encode the respective biologically functional molecules in *T. hemsleyanum*, in contrast with grapes, with two copies of each gene [20]. This also further hinted that the regulatory pathway of PA in *T. hemsleyanum* might be more complicated than in the grape, as there were more genes involved in the biosynthesis of PA (Figure 6 and Figure 8), according to the PPI network. 

As a source of nutrition, energy, and medicine for humans, either directly or indirectly, plants can synthesize a large number of metabolites with different biological functions, especially Chinese herbal plants, as is well known, their metabolites are vitally important for human health. The commonly used Chinese herbal *T. hemsleyanum* contains a large number of secondary metabolites. Its root tuber is used as the main part of some medicines, and it has a unique curative effect on various fevers and edema [3]. Currently, flavonoids, phenolic substances, triterpenoids, steroids, and stilbene compounds have been found in *T. hemsleyanum*. Metabolomics research utilizes liquid chromatography tandem mass spectrometry (LC-MS/MS) to accurately identify and quantify active components—that is, to detect and screen out biologically and statistically significant metabolite active components from PL and RG ecotypes. The mainly differential components include flavonoids, flavonols, catechins, anthocyanins, Pas, and organic acids (Figure 1 and Figure 2, Table S1). Therefore, it is challenging to unravel which substances play the leading role in treating diseases. As a member of the polyphenol family, PAs have strong antioxidant activities and the newest published study revealed that the procyanidin C1 from a polyphenolic component of grape seed extract had senotherapeutic activity and increases lifespan in mice [40]. Since the procyanidins A3, B2, and B3 are enriched in PLs, their functions provide a major study arena for research in *T. hemsleyanum*. Therefore, future research should focus on rationally developing and utilizing these wild medicinal plant resources. 

## 4. Materials and Methods

### 4.1. Plant Materials and Growth Distribution, Collection Location

The rapid growth green (RG) and purple leaf (PL) genotypes of *Tetrastigma hemsleyanum* were collected from Guilin, Guangxi (25°36′24′′ N; 109°54′20′′ E) and Huaihua, Hunan (27°31′12′′ N; 110°6′36′′ E) provinces, respectively. Subsequently, the collected wild resources were cultivated in the greenhouse of Hangzhou Academy of Agricultural Sciences (Hangzhou, Zhejiang Province, China) as materials for research. 

### 4.2. Samples Library Preparation for Transcriptome Sequencing 

Leaves at the third node away from the top of the plants (PL and RG) were collected and frozen in liquid nitrogen and stored at −80°C for RNA-seq. At least 1 μg RNA per sample was used as input material for the RNA sample preparations. RNA concentration was measured using NanoDrop 2000 (Thermo Fisher Scientific Inc., Waltham, MA, USA). RNA integrity was assessed using the RNA Nano 6000 Assay Kit of the Agilent Bioanalyzer 2100 system (Agilent Technologies, Santa Clara, CA, USA). Sequencing libraries were generated using NEBNext^®^ Ultra™ RNA Library Prep Kit for Illumina^®^ (NEB, Ipswich, MA, USA) following the manufacturer’s recommendations, and index codes were added to attribute sequences to each sample. Briefly, mRNA was purified from the total RNA using poly-T oligo-attached magnetic beads. Fragmentation was carried out using divalent cations under elevated temperature in NEBNext^®^ First Strand Synthesis Reaction Buffer (5×). First strand cDNA was synthesized using a random hexamer primer and M-MuLV Reverse Transcriptase. Second strand cDNA synthesis was subsequently performed using DNA Polymerase I and RNase H. The remaining overhangs were converted into blunt ends via exonuclease/polymerase activities. After adenylation of the 3′ ends of the DNA fragments, a NEBNext^®^ Adaptor with hairpin loop structure was ligated to prepare for hybridization. In order to select cDNA fragments of preferentially 240 bp in length, the library fragments were purified with an AMPure XP system (Beckman Coulter, Beverly, CA, USA). Then, 3 μL USER^®^ Enzyme (NEB, USA) was used with size-selected, adaptor-ligated cDNA at 37 °C for 15 min followed by 5 min at 95 °C before PCR. Then, PCR was performed with Phusion^®^ High-Fidelity DNA polymerase, Universal PCR primers and Index (X) Primer. Lastly, the PCR products were purified (AMPure XP system) and the library quality was assessed using the Agilent Bioanalyzer 2100 system. The heat map of PA metabolic related differential gene expressions was made by TB tools [41].

#### 4.2.1. Gene Functional Annotation

Gene function was annotated based on the following databases: NR (NCBI non-redundant protein sequences); Pfam (Protein family); KOG/COG/eggNOG (Clusters of Orthologous Groups of proteins); Swiss-Prot (a manually annotated and reviewed protein sequence database); KEGG (Kyoto Encyclopedia of Genes and Genomes); GO (Gene Ontology). 

#### 4.2.2. KEGG Pathway Enrichment Analysis

KEGG (Kanehisa et al., 2008) is a database resource for understanding high-level functions and utilities of the biological system, such as the cell, the organism, and the ecosystem, from molecular-level information, especially large-scale molecular datasets generated by genome sequencing and other high-throughput experimental technologies (http://www.genome.jp/kegg/, accessed on 8 January 2022). We used KOBAS [42] software to test the statistical enrichment of differential expression genes in KEGG pathways.

#### 4.2.3. Protein–Protein Interaction Networks

The sequences of the DEGs were blast (blastx) to the genome of a related species (the protein–protein interaction of which exists in the STRING database: http://string-db.org/, accessed on 8 January 2022) to get the predicted PPI of these DEGs. Then, the PPI of these DEGs was visualized in Cytoscape [43]. 

### 4.3. Metabolic Profiling

#### 4.3.1. Sample Preparation and Extraction

The freeze-dried leaf was crushed using a mixer mill (MM 400, Retsch) with a zirconia bead for 1.5 min at 30 Hz. Then, 100 mg powder was weighted and extracted overnight at 4 °C with 1.0 mL 70% aqueous methanol. Following centrifugation at 10,000× *g* for 10 min, the extracts were absorbed (CNWBOND Carbon-GCB SPE Cartridge, 250 mg, 3 mL; ANPEL, Shanghai, China, www.anpel.com.cn/cnw, accessed on 8 January 2022) and filtrated (SCAA-104, 0.22 μm pore size; ANPEL, Shanghai, China, http://www.anpel.com.cn/, accessed on 8 January 2022) before LC-MS analysis.

#### 4.3.2. HPLC Conditions

The sample extracts were analyzed using an LC-ESI-MS/MS system (HPLC, Shim-pack UFLC SHIMADZU CBM30A system, www.shimadzu.com.cn/, accessed on 8 January 2022; MS, Applied Biosystems 6500 Q TRAP, www.appliedbiosystems.com.cn/, accessed on 8 January 2022). The analytical conditions were as follows, HPLC: column, Waters ACQUITY UPLC HSS T3 C18 (1.8 µm, 2.1 mm × 100 mm); solvent system, water (0.04% acetic acid): acetonitrile (0.04% acetic acid); gradient program, 95:5 *v*/*v* at 0 min, 5:95 *v*/*v* at 11.0 min, 5:95 *v*/*v* at 12.0 min, 95:5 *v*/*v* at 12.1 min, 95:5 *v*/*v* at 15.0 min; flow rate, 0.40 mL/min; temperature, 40 °C; injection volume: 2 μL. The effluent was alternatively connected to an ESI-triple quadrupole-linear ion trap (Q TRAP)-MS. 

#### 4.3.3. ESI-Q TRAP-MS/MS

LIT and triple quadrupole (QQQ) scans were acquired on a triple quadrupole-linear ion trap mass spectrometer (Q TRAP), API 6500 Q TRAP LC/MS/MS System, equipped with an ESI Turbo Ion-Spray interface, operating in a positive ion mode and controlled by Analyst 1.6.3 software (AB Sciex). The ESI source operation parameters were as follows: ion source, turbo spray; source temperature 500 °C; ion spray voltage (IS) 5500 V; ion source gas I (GSI), gas II (GSII), the curtain gas (CUR) settings were set at 55, 60, and 25.0 psi, respectively; the collision gas (CAD) was high. Instrument tuning and mass calibration were performed with 10 and 100 μmol/L polypropylene glycol solutions in QQQ and LIT modes, respectively. QQQ scans were acquired as MRM experiments with the collision gas (nitrogen) set to 5 psi. DP and CE for individual MRM transitions was done with further DP and CE optimization. A specific set of MRM transitions were monitored for each period according to the metabolites eluted within this period.

### 4.4. DMACA Staining of PAs in RG and PL Leaves 

The RG and PL leaves originated from different habitats as experimental materials; the qualitative analysis of PAs were carried out according to a modified previously published study method [44]. Dimethylaminocinnamaldehyde (DMACA) staining was performed using fresh *T. hemsleyanum* leaves soaked in 1% DMACA solution (1% [*w*/*v*] ethanol solution: 6 N HCl, 1:1 [*v*/*v*]) overnight followed by washing 3 times with 70% ethanol. A digital camera (Canon, Japan) was used to photograph pictures of the stained leaves.

## 5. Conclusions

The purpose of this study was to reveal the accumulation characteristics of PA synthesis and find the key genes related to PA synthesis. We found that hundreds of differential metabolites were screened out, and, in particular, two types of PA were highly accumulated in the leaves of the PL ecotypes of *T. hemsleyanum*, namely A-type and B-type PA, but not in the RG ecotype. The RNA-seq analysis showed that the high expression of DFR1/2 and its downstream gene ANS in PL might promote the accumulation of LAs and anthocyanins. Since ANR and LAR are pivotal genes involved in the PA pathway, their high expression is the main factor that causes the accumulation of PAs. It can, thus, be seen that there may exist more complicated regulatory pathways for the synthesis of PA in *T. hemsleyanum* than in grapes, according to the protein–protein interaction network. This was the first time PA metabolism has been analyzed at the genetic and metabolome level. The role of time and tissue-specific regulation of plant genes in the activity of traditional Chinese medicines is a very important area for further exploration, particularly for future attempts to change the biosynthesis of PA in traditional Chinese medicine herb leaves. This study of the anabolic mechanism of PA will help the excavation and utilization of the active ingredients of traditional Chinese medicine herbs.

## Data Availability

The data presented in this study are available within the article and its Supplementary Materials.

## Supplementary Materials

The following are available online at https://www.mdpi.com/article/10.3390/plants11020211/s1, Table S1: Differential genes and metabolites between PL and RG.
